# Novel extracellular and nuclear caspase-1 and inflammasomes propagate inflammation and regulate gene expression: a comprehensive database mining study

**DOI:** 10.1186/s13045-016-0351-5

**Published:** 2016-11-14

**Authors:** Luqiao Wang, Hangfei Fu, Gayani Nanayakkara, Yafeng Li, Ying Shao, Candice Johnson, Jiali Cheng, William Y. Yang, Fan Yang, Muriel Lavallee, Yanjie Xu, Xiaoshu Cheng, Hang Xi, Jonathan Yi, Jun Yu, Eric T. Choi, Hong Wang, Xiaofeng Yang

**Affiliations:** 1 Centers for Metabolic Disease Research, Lewis Katz School of Medicine at Temple University, 3500 North Broad Street, MERB-1059, Philadelphia, PA 19140 USA; 2 Cardiovascular Research and Thrombosis Research, Lewis Katz School of Medicine at Temple University, 3500 North Broad Street, MERB-1059, Philadelphia, PA 19140 USA; 3 Department of Pharmacology, Lewis Katz School of Medicine at Temple University, 3500 North Broad Street, MERB-1059, Philadelphia, PA 19140 USA; 4 Department of Physiology, 3500 North Broad Street, MERB-1059, Philadelphia, PA 19140 USA; 5 Department of Surgery, Lewis Katz School of Medicine at Temple University, 3500 North Broad Street, MERB-1059, Philadelphia, PA 19140 USA; 6 Department of Cardiovascular Medicine, the Second Affiliated Hospital of Nanchang University, 1 Minde Road, Nanchang, Jiangxi 330006 China

**Keywords:** Caspase-1, Trafficking, Nuclear gene regulation, Inflammation propagation, Exosome

## Abstract

**Background:**

Caspase-1 is present in the cytosol as an inactive zymogen and requires the protein complexes named “inflammasomes” for proteolytic activation. However, it remains unclear whether the proteolytic activity of caspase-1 is confined only to the cytosol where inflammasomes are assembled to convert inactive pro-caspase-1 to active caspase-1.

**Methods:**

We conducted meticulous data analysis method﻿s on proteomic, protein interaction, protein intracellular localization, and gene expressions of 114 experimentally identified caspase-1 substrates and 38 caspase-1 interaction proteins in normal physiological conditions and in various pathologies.

**Results:**

We made the following important findings: (1) Caspase-1 substrates and interaction proteins are localized in various intracellular organelles including nucleus and secreted extracellularly; (2) Caspase-1 may get activated in situ in the nucleus in response to intra-nuclear danger signals; (3) Caspase-1 cleaves its substrates in exocytotic secretory pathways including exosomes to propagate inflammation to neighboring and remote cells; (4) Most of caspase-1 substrates are upregulated in coronary artery disease regardless of their subcellular localization but the majority of metabolic diseases cause no significant expression changes in caspase-1 nuclear substrates; and (5) In coronary artery disease, majority of upregulated caspase-1 extracellular substrate-related pathways are involved in induction of inflammation; and in contrast, upregulated caspase-1 nuclear substrate-related pathways are more involved in regulating cell death and chromatin regulation.

**Conclusions:**

Our identification of novel caspase-1 trafficking sites, nuclear and extracellular inflammasomes, and extracellular caspase-1-based inflammation propagation model provides a list of targets for the future development of new therapeutics to treat cardiovascular diseases, inflammatory diseases, and inflammatory cancers.

**Electronic supplementary material:**

The online version of this article (doi:10.1186/s13045-016-0351-5) contains supplementary material, which is available to authorized users.

## Background

As a member of the cysteinyl aspartate-specific protease caspase family, caspase-1 is present in the cytosol as pro-caspase-1, an inactive zymogen, and requires the assembly of cytosolic multi-protein complexes known as “inflammasomes” for proteolytic activation [1]. These complexes are assembled intracellularly in response to damage/danger signal-associated molecular patterns (DAMPs) and pathogen-associated molecular patterns (PAMPs), similar to the role of Toll-like receptors (TLRs) at the cell surface [2]. Activated caspase-1 is required for cleaving/processing of pro-interleukin-1β (pro-IL-1β) and pro-IL-18 into mature pro-inflammatory cytokines IL-1β and IL-18 in the cytosol, respectively. Additionally, activated caspase-1 induces other inflammatory pathways by degrading anti-inflammatory sirtuin-1 (Sirt-1), a nicotinamide adenine dinucleotide (NAD)-dependent protein/class III histone deacetylase [3]. Caspase-1 has been shown to induce cell necrosis, pyroptosis, or pyrop-apoptosis [4] and play a significant role in various developmental stages [5]. However, most of the biological activities of caspase-1 reported so far take place at the post-translational level (proteolytic processing). Moreover, it remains unclear whether all the substrates of caspase-1 are only localized in the cytosol where inflammasomes are assembled to convert inactive pro-caspase-1 to active caspase-1.

The regulatory effects of caspase-1 on gene expression have been reported in the intestine [6], liver [6], and adipose tissue [7]. In addition, we also have reported several significant findings on the expression and roles of caspase-1 in vascular inflammation: (1) Nod (nucleotide-binding and oligomerization domain)-like receptors, inflammasome components, and caspases are differentially expressed in human and mouse tissues [8]; (2) Caspase-1 can recognize extended cleavage sites on its natural substrates [9]; (3) Early hyperlipidemia promotes endothelial activation/dysfunction [10] via a caspase-1–Sirt-1 pathway [11]; (4) Inhibition of caspase-1 activation improves angiogenesis [12]; (5) Caspase-1 weakens the progenitor cell [13] -mediated vessel repair [14] in hyperlipidemia; (6) Caspase-1 mediates chronic kidney disease-promoted neointima hyperplasia of the carotid artery [15], and (7) Hyper-homocysteinemia, an independent risk factor for cardiovascular disease, induces caspase-1-mediated pyrop-apoptosis [4].

Despite all these findings of biological functions of caspase-1, it is not clear whether the caspase-1 activity is limited only to the cytosol. Variety of inflammasome assemblies are mainly recognized to be functional in the cytosol [16]. In addition to well-characterized caspase-1 substrates interleukin-1β (IL-1β) and IL-18 [17], we and others previously reported that caspase-1 cleaves Sirt-1, a histone deacetylase, predominantly found in the nucleus, which is a clear indication that the function of caspase-1 may extend to other subcellular compartments such as nucleus [3, 11]. Similarly, as we previously reported, caspase-1 (enzyme ID: EC 3.4.22.36) cleaves many other protein substrates including those listed in the Brenda enzyme database [9, 18], and nuclear transcription factors such as GATA-Binding Protein 4 (GATA4) [19] and peroxisome proliferator-activated receptor γ (PPARγ) [20]. This provides further validation that caspase-1 and its inflammasome components may play a role in intracellular compartments other than the cytosol.

**Fig. 1:**
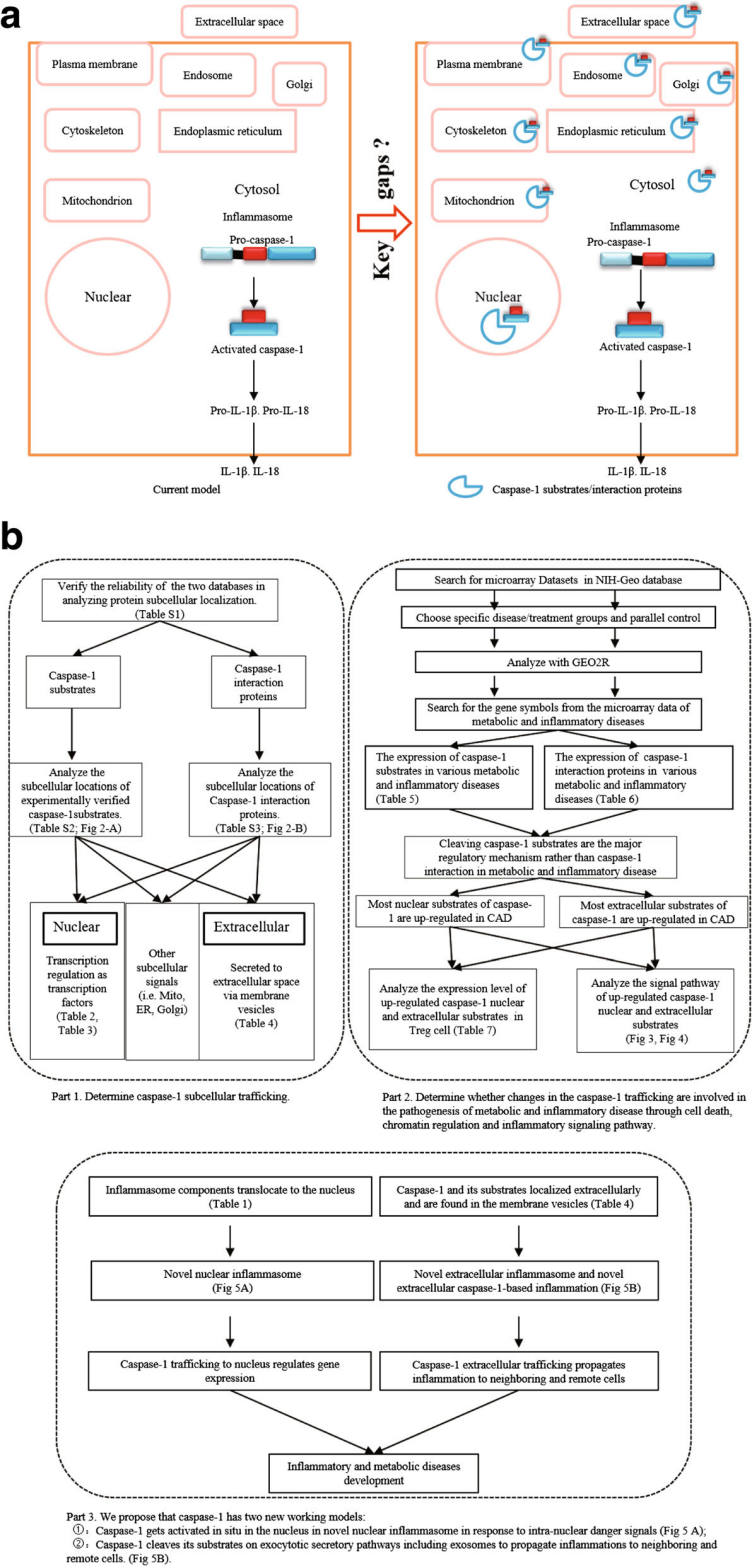
The key knowledge gaps of the current caspase-1 model and the flow chart of our database mining strategy. **a** The key knowledge gaps between the current model and the newly proposed caspase-1 trafficking model. The new model suggests that caspase-1 may traffic to various subcellular organelles to interact and cleave its substrates and regulate variety of biological functions. **b** Flow chart of database mining strategy and three parts of data organization. We propose caspase-1 has two new working models: (1) caspase-1 gets activated in situ in the nucleus as novel nuclear inflammasome in response to intra-nuclear danger signals; (2) caspase-1 cleaves its substrates in exocytotic secretory pathways including exosomes to propagate inflammation to neighboring and remote cells

In supporting our argument, a recent report showed that a well-established component of inflammasome complex called NLRP3/NALP3 [NLR (nucleotide-binding oligomerization domain-like (Nod)-like receptor) family pyrin domain containing 3] acts as a transcription regulator of type 2 T helper cell (Th2) differentiation [21]. NLRP3 is a well-characterized NLR and contains NACHT [a domain present in NAIP (neuronal apoptosis inhibitor protein), C2TA (MHC class 2 transcription activator), HET-E (incompatibility locus product from *Podospora anserina*), and TP-1 (telomerase protein-1)], LRR (leucine-rich repeat), and PYD (pyrin) domains. Moreover, it was reported that caspase-1 can be activated in the nucleus [22, 23], suggesting the possibility of caspase-1 being a direct regulator of gene expression. In addition to regulating non-classic secretory pathway [24], caspase-1 is also found in extracellular secretory exosomes, which further prove that caspase-1 is functional outside the cytosol. Together with our findings and others’, we hypothesized that caspase-1 play a dynamic role by trafficking to various subcellular organelles and regulate variety of biological functions by interacting and cleaving its protein substrates (Fig. 1a).

Since previous reports showed that enzymatic activities reflect the subcellular localization of enzyme [25] and that protein localization features can also predict protein functions [26], we conducted an extensive analysis on reported data to test our hypothesis. We have included our data mining strategy which we have utilized in our previous publications in the “Methods” section. Our comprehensive analysis yielded the following significant and novel findings of caspase-1: (1) intracellular localization of its substrates and interaction proteins indicate that caspase-1 traffics extensively to various intracellular organelles including mitochondria, nucleus, and extracellular space; (2) 7 out of 27 caspase-1 nuclear substrates and 3 of 7 nuclear interaction proteins have transcription regulatory functions, suggesting that caspase-1 regulates gene transcription; and (3) caspase-1, 23 caspase-1 substrates, and 2 caspase-1 interaction proteins are secreted extracellularly, suggesting the possibility of caspase-1-mediated propagation of inflammation from cell to cell or to remote cells via circulation. Our findings may eventually lead to future development of novel therapeutics for the treatment of chronic sterile inflammations such as cancers and cardiovascular diseases.

## Methods

### Search for the intracellular localization of caspase-1 substrates and caspase-1 interaction proteins

We have utilized a novel approach which is illustrated in Fig. 1b. We analyzed 114 experimentally verified caspase-1 substrates that were updated in recently published reports and review [9, 27, 28]. These substrates were identified by utilizing proteomic approaches. In addition, we examined 38 experimentally identified human caspase-1 interaction proteins reported in the NIH-NCBI-Gene database [29]. All of the caspase-1 interaction proteins were experimentally verified in previous publications listed in PubMed (all the references are included in Additional file 1: Table S3). The identification methods that were used in these publications included biochemical activity, affinity capture-mass spectrometry, affinity Capture-Western blot, two hybrid systems, and reconstituted complex.

Then we analyzed the subcellular localization of these experimentally verified caspase-1 substrates and interaction proteins in two widely used protein intracellular localization databases named as Compartments subcellular location database [30] and UniProtKB/Swiss-Prot location database (European Bioinformatics Institute) [31]. To demonstrate the reliability of these databases, subcellular localization of 21 generally accepted intracellular organelle markers were analyzed and presented in Additional file 1: Table S1. This table includes the PubMed IDs of publications where these organelle markers were experimentally utilized, which implicates that the two database sources that we used are extremely reliable to predict the subcellular localizations of proteins of interest.

**Table 1:** 6/20 NLRs are localized in nucleus, suggesting assembly of a variety of nuclear inflammasomes. Several NLR proteins have potential to form nuclear inflammasome with caspase-1

Symbol	Source	Subcellular localizations (probabilities)				
		Database 1^a^			Database 2^b^	Summary
		High	Middle	Low		
NOD1	1	PM	Cytosol	Nucleus	Cytoplasm	Cytoplasm
NOD2	1	Cytoskeleton	PM	Cytosol	Cytoplasm	Cytoplasm
NOD3	1	Cytoplasm			Cytoplasm	Cytoplasm
NOD4	1	Cytosol	Nucleus	Cytoskeleton	Cytoplasm	Cytoplasm
NALP1	1	Cytosol	Nucleus		Nucleus	Nucleus
NALP2	1	Cytosol			Nucleus	Cytoplasm
NALP3	1	Cytosol	Extracellular	Nucleus	Nucleus	Nucleus
NALP4	1	Extracellular	Cytosol			Extracellular
NALP5	1	Nucleus	Mito	Cytosol	Nucleus	Nucleus
NALP6	1	Nucleus	PM	Cytosol	Nucleus	Nucleus
NALP7	1	Cytosol				Cytosol
NALP9	1	Cytoplasm			Cytoplasm	Cytoplasm
NALP10	1	PM	Nucleus	Cytosol	Cytoplasm	PM
NALP12	1	Cytosol			Cytoplasm	Cytosol
NALP14	1	Cytosol				Cytosol
NAIP	1	Extracellular	PM	Nucleus		Extracellular
IPAF	1	Cytosol	Nucleus	Cytoskeleton	Cytosol	Cytosol
IFI16	2	Nucleus	Cytosol		Nucleus	Nucleus

*Abbreviation*: *1* from published paper (PMID:19505385), *2* from published paper (PMID:25466628), *PM* plasma membrane, *ER* endoplasmic reticulum, *Mito* mitochondrion

^a^Compartments subcellular location database

^b^UniProtKB/Swiss-Prot location database

*Candidate nuclear inflammasome molecules

**Table 2:** Pro-caspase-1 and inflammasome components traffic into nucleus and form active inflammasomes that activate caspase-1 in situ

Inflammasome components		Function	Location	Translocation	PMID
ASC	ASC-b	Inflammasome adaptor	Cytoplasm	Nucleus	20482797
	ASC-c	Inflammasome adaptor	Cytoplasm	Nucleus	
	ASC-d		Cytosol	Nucleus	
Pro-caspase-1		Pro-caspase-1 and caspase prodomain promote translocation	Cytosol	Nucleus	9726961
NLRP3		Nuclear DNA-binding transcription factor	Cytosol	Nucleus	26098997
NLRA		Control transcription, inflammasome formation and transcriptional activity		Nucleus	26194278
NLRC5		Control transcription, inflammasome formation and transcriptional activity		Nuclear	26194278

*Abbreviation: ASC* apoptosis-associated speck-like protein containing a carboxy-terminal CARD, *NLR* nucleotide-binding oligomerization domain-like (Nod)-like receptor, *PMID* PubMed identifier

### Expression profile of caspase-1 substrates and interaction proteins in human disease, and mouse disease models

Gene expression profile of identified caspase-1 substrates and its interaction proteins were analyzed in 13 microarray datasets extracted from NIH-GEO database (Fig. 1b). Specific samples were chosen as disease or treatment groups and parallel controls. The number of samples was always greater than three except for the pooled samples. First, we selected the genes with significant expression changes (*p* < 0.05) in the microarray data set and examined the fold change of the genes of our interest. The genes with more than onefold expression change were defined as the upregulated genes while genes with their expression changes less than onefold were defined as downregulated genes.

### Ingenuity Pathway Analysis

We utilized Ingenuity Pathway Analysis (IPA, Ingenuity Systems, www.ingenuity.com) to characterize clinical relevance and molecular and cellular functions related to the identified genes in our microarray analysis. The differentially expressed genes were identified and uploaded into IPA for analysis. The core and pathways analysis was used to identify molecular and cellular pathways.

## Results

### Caspase-1 substrates are localized in various intracellular organelles including nucleus and also secreted extracellularly

The well-accepted working model of caspase-1 is that the dormant pro-caspase-1 undergoes self-cleavage and becomes activated in the cytosolic protein complex termed inflammasome, which is assembled in response to external and internal danger signals. Once being activated, caspase-1 gains its ability to cleave its substrates; therefore, it cleaves cytosolic pro-IL-1β and pro-IL-18 into mature IL-1β and IL-18 respectively. Then these cytokines get secreted extracellularly to propagate inflammation. Therefore, we reasoned that if the current model is valid, all the caspase-1 substrates should be localized in the cytosol in order to get access to caspase-1-mediated cleavage. However, recent reports from our lab and others’ challenged this working model. We and others demonstrated that caspase-1 cleaves Sirt-1 [3, 10], which is a nuclear localized histone deacetylase. Moreover, caspase-1-mediated cleavage of nuclear transcription factors such as GATA-Binding Protein 4 (GATA4) [19] and peroxisome proliferator-activated receptor γ (PPARγ) [20] was also reported. Thus, we hypothesized that caspase-1 can traffic to subcellular locations outside the cytosol to fulfill its catalytic functions.

To examine this hypothesis, we analyzed the subcellular localization of caspase-1 substrates and its interaction proteins in widely used databases named Compartments subcellular location database and UniProtKB/Swiss-Prot location database. We tested the reliability of these databases by identifying the intracellular localization of experimentally verified 21 well-known organelle marker proteins. In our test, we included two plasma membrane proteins, two cytosolic proteins, two endosome proteins, three endoplasmic reticulum (ER) proteins, three Golgi proteins, three mitochondrial proteins, three cytoskeletal proteins and three nuclear proteins (Additional file 1: Table S1). We found that all of the intracellular organelle marker proteins are identified in these two databases to be localized in their intrinsic organelles as reported (see the PubMed IDs of the related papers), suggesting that these two protein intracellular localization databases are highly reliable.

**Fig. 2:**
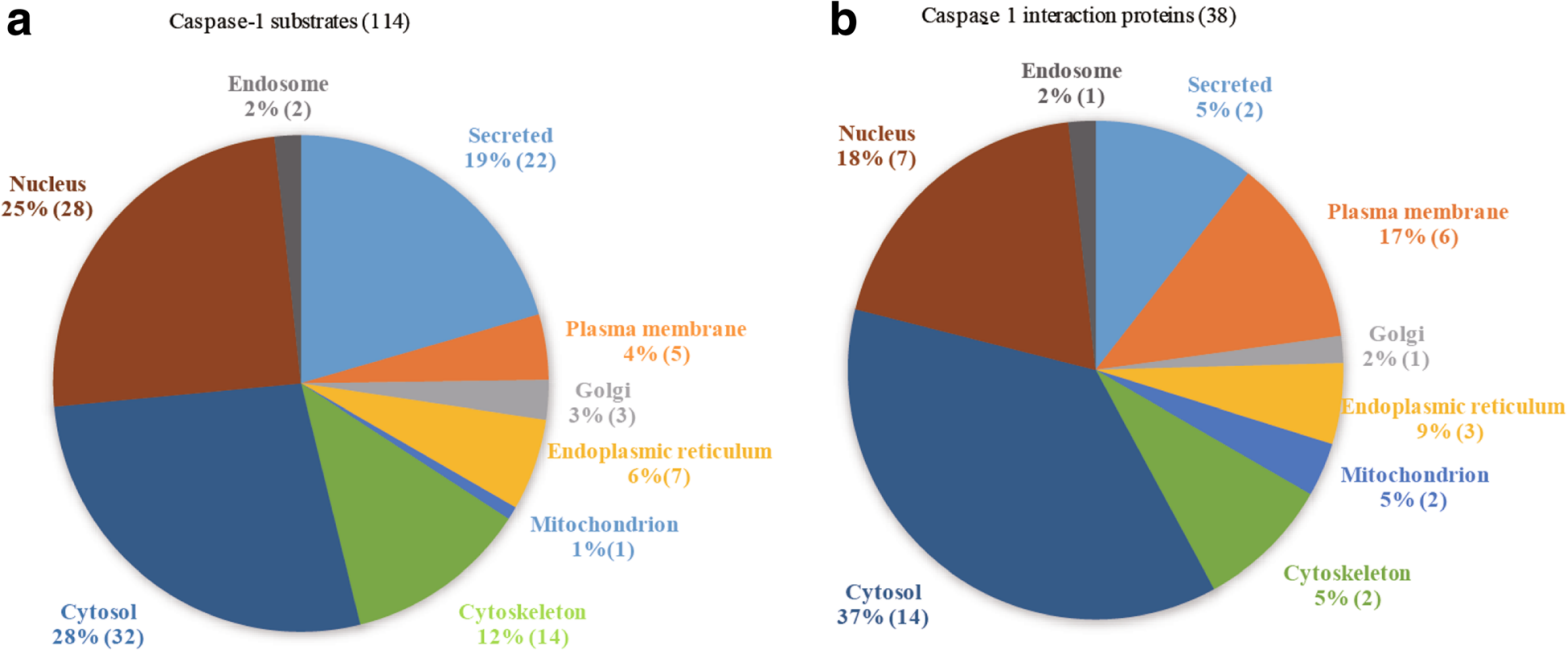
Caspase-1 substrates and interaction proteins are present in cellular compartments other than the cytosol. **a** There are 114 newly experimentally verified caspase-1 substrates: 22 of these caspase-1 substrates are secretory proteins, 5 are plasma membrane proteins, 3 Golgi proteins, 7 ER proteins, 1 mitochondrial protein, 14 cytoskeleton proteins, 33 cytosolic proteins, 28 nuclear proteins, and 2 endosomal proteins. 28% of the identified caspase-1 substrates are present in the cytosol while the rest of the 72% of the substrates are generally localized elsewhere other than in the cytosol. 25% caspase-1 substrates are localized in the nucleus and 19% caspase-1 substrates are secreted extracellularly. **b** Out of 38 experimentally verified caspase-1 interaction proteins, 6 caspase-1 interaction proteins are secretory proteins, 7 are plasma membrane proteins, 1 Golgi protein, 3 ER proteins, 2 mitochondrial proteins, 2 cytoskeleton proteins, 18 cytosolic proteins, 11 nuclear proteins, and 1 endosomal protein. In addition to 35% cytosolic interaction proteins, caspase-1 has as high as 63% probabilities to traffic to other subcellular organelles to form protein interactions. 21% of caspase-1 interaction proteins are localized in the nucleus and 11% of caspase-1 interaction proteins are secreted extracellularly

Then, we analyzed the intracellular localization of 114 newly experimentally verified caspase-1 substrates (Additional file 1: Table S2) and found that 22 of these caspase-1 substrates are secretory proteins, 5 are plasma membrane proteins, 3 Golgi proteins, 7 ER proteins, 1 mitochondrial protein, 14 cytoskeleton proteins, 32 cytosolic proteins, 28 nuclear proteins, and 2 endosomal proteins. The results suggested that 28% of the identified caspase-1 substrates are present in the cytosol and comply well with the current accepted model of caspase-1 while rest of the 72% of the substrates are generally localized elsewhere other than in the cytosol. Most surprisingly, 25% caspase-1 substrates are localized in the nucleus and 19% caspase-1 substrates are secreted extracellularly. This clearly suggested that the current model cannot be applied to majority of experimentally verified caspase-1 substrates as there is a 72% probability that caspase-1 may traffic to other subcellular organelles to exert its catalytic functions (Fig. 2a).

### Caspase-1 interaction proteins are localized in various intracellular organelles including nucleus and secreted extracellularly

To consolidate our finding that the majority of caspase-1 substrates are localized in subcellular compartments other than the cytosol, we analyzed whether the caspase-1-interacting proteins that are required for caspase-1 function are also found in these subcellular domains. We reasoned that if the current model is valid, all the experimentally validated caspase-1 interaction proteins should be localized in the cytosol in order to get access to caspase-1 for interaction. Although various types of protein interactions share the same sets of interacting bonds, enzyme-substrate interaction may additionally require accurate binding orientation to enzyme active site for catalytic activities [32].

As elaborated in the “Methods” section, we identified 38 experimentally verified caspase-1 interaction proteins and analyzed their intracellular localization (Additional file 1: Table S3). Our results indicated that 2 caspase-1 interaction proteins are secretory proteins, 6 are plasma membrane proteins, 1 Golgi protein, 3 ER proteins, 2 mitochondrial proteins, 2 cytoskeleton proteins, 14 cytosolic proteins, 7 nuclear proteins, and 1 endosomal protein. Once again, the results derived from the analysis of the intracellular localization of caspase-1 interaction proteins suggest that, in addition to 37% cytosolic interaction proteins that correlated well with the current model, caspase-1 has as high as 63% probability to traffic to other subcellular organelles to exert its functions by protein interactions (Fig. 2b). Interestingly, an 18% of caspase-1 interaction proteins are localized in the nucleus while 5% of caspase-1 interaction proteins are secreted extracellularly.

### Caspase-1 gets activated in situ in the nucleus in response to intra-nuclear danger signals

Recently, we and others found that a histone deacetylase Sirt-1, a protein predominantly localized in the nucleus as suggested by the GeneCards database [33], is specifically cleaved by caspase-1 in human aortic endothelial cells in response to pro-atherogenic stimuli such as oxidized low density lipoprotein (oxLDL) [11]. Further, caspase-1 cleaves Sirt-1 in apolipoprotein E-deficient (ApoE^−/−^) mouse aorta in response to high-fat diet feeding [11], and in adipose tissue in response to high-fat diet-induced metabolic dysfunction [3]. In addition, protein nuclear transcription factors such as GATA-Binding Protein 4 (GATA4) [19] and peroxisome proliferator-activated receptor γ (PPARγ) [20] were also found to be caspase-1 substrates.

These findings raise an important question to be addressed, that is whether pro-caspase-1 traffics to the nucleus and get activated in the nucleus or activated caspase-1 traffics to the nucleus. A previous report by *Mao et al.* showed that tumor necrosis factor-α (TNF-α) induces apoptosis by triggering trafficking of pro-caspase-1, but not activated caspase-1, into the nucleus and getting caspase-1 activated in situ [22]. Since pro-caspase-1 requires assembly of inflammasomes that consists of two other proteins [17] including a nucleotide-binding oligomerization domain-like (Nod)-like receptor (NLR) and apoptosis-associated speck-like protein containing a carboxy-terminal CARD (ASC), we hypothesized that in order to get pro-caspase-1 activated in the nucleus, these inflammasome components should also be localized in the nucleus as well. In supporting our argument, a recent report showed that NLRP3/NALP3, a well-characterized NLR, is a nuclear transcription regulator of type 2 T helper cell (Th2) differentiation [21]. Moreover, it was also reported that ASC is predominantly localized in the nucleus in resting human monocytes/macrophage and is trafficked to cytosol to form an active inflammasome in response to pathogen infection [34]. All these findings suggest the possibility of the presence of NLRP3 inflammasome in the nucleus.

There are about 20 NLRs expressed in human genome [8]; therefore, we analyzed the subcellular localization of all the 20 NLRs. As shown in Tables 1 and 2, six out of 20 NLRs examined including NALP1, NALP3, NALP5, NALP6 (Table 1), NLRA, and NLRC5 (Table 2) are localized in the nucleus. Since an inflammasome is composed of one type of NLR, ASC, and pro-caspase-1, our results suggested that several types of nuclear inflammasomes can be assembled to get caspase-1 activated in the nucleus for regulating inflammation. Our argument is supported by a recent report by *Kerur et al.*, who demonstrated that during Kaposi sarcoma-associated herpes virus infection in endothelial cells, interferon gamma-inducible protein 16 (IFI16) interacts with the adaptor molecule ASC and pro-caspase-1 to form a functional nuclear inflammasome [35]. IFI16 (NCBI Protein database ID: NP_001193496) has 729 amino acids and is a member of the HIN-200 (hematopoietic interferon-inducible nuclear antigens with 200 amino acid repeats) family of cytokines. IFI16 has also caspase activation and recruitment domain CARD and pyrin domain [36], which is functionally similar to other NLRs. The nuclear inflammasomes may be functional for sensing nuclear danger signals including DNA virus infections [35], TNF-α induced apoptosis [22] (DNA fragmentation), GATA4 [19] -mediated cardiac development, and PPARγ [20] -mediated adipocyte differentiation, and enhancing insulin resistance [37], Sirt-1 degradation-triggered pro-inflammatory process [11], and other genome regulation dysfunctions [38]. We have summarized the nuclear danger signals reported so far in Additional file 1: Table S4. In addition, our results also showed that 6 out of 27 caspase-1 nuclear substrates (Table 3) and 3 of 7 interaction proteins (Table 4) have transcription regulatory functions, suggesting that caspase-1 is a potential gene regulator.

**Table 3:** 6/27 caspase-1 nuclear substrates have transcription regulatory functions

Substrate	Location summary	Transcription factor		Inflammation	
		PAZAR TF (ID)	NCBI	Inflammatory function	PMID
HSPB3	Nucleus				
HNRNPA2B1	Nucleus			Pro-inflammation	26030368
SMG7	Nucleus			Pro-inflammation	21467779
TFAP2A	Nucleus	TF0000385	^a^	Pro-inflammation	10504447
PTPN18	Nucleus				
MCM3	Nucleus			Pro-inflammation	12421976
VPS72	Nucleus				
EEF1A1	Nucleus	TF0000325		Pro-inflammation	22829547
LMNA	Nucleus			Pro-inflammation	18551513
GIT2	Nucleus			Pro-inflammation	16715100
PCBP2	Nucleus			Pro-inflammation	19740317
PARP1	Nucleus			Pro-inflammation	17430886
ATXN3	Nucleus			Pro-inflammation	11466410
U2AF2	Nucleus				
HTATSF1	Nucleus		^a^	Pro-inflammation	21830069
BIRC4	Nucleus				
MATR3	Nucleus				
NAV3	Nucleus				
ASCC2	Nucleus				
SCAF11	Nucleus				
TUB	Nucleus		^a^		
ZC3HAV1	Nucleus				
ZMAT2	Nucleus				
PPARG	Nucleus	TF0000041	^a^	Pro-inflammation	11089900
NONO	Nucleus				
MCM5	Nucleus			Anti-inflammation	10551502
TRIM28	Nucleus	TF0000281	^a^	Anti-inflammation	22995936

*Abbreviation*: *PAZAR TF* Transcription factor database (http://www.pazar.info/cgi-bin/tf_search.cgi), *NCBI* National Center of Biotechnology Information, *PMID* PubMed identifier

^a^Genes are confirmed in NCBI

**Table 4:** 3/7 caspase-1 interaction proteins have transcription regulatory functions

CASP1 interaction proteins	Location summary	Transcription factor
		NCBI
AR	Nucleus	^a^
ATN1	Nucleus	^a^
CDK11A	Nucleus	
CDK11B	Nucleus	
ARID4B	Nucleus	
BIRC3	Nucleus	
CEBPB	Nucleus	^a^

*Abbreviation*: *CASP1* caspase-1, *NCBI* National Center of Biotechnology Information

^a^Genes are confirmed in NCBI

### Caspase-1 cleaves substrates on exocytotic secretory pathways to propagate inflammation to neighboring and remote cells

Current understanding on the cytosolic roles of caspase-1 in promoting inflammation is based on the following findings: (1) processing pro-IL-1β and pro-IL-18 into mature IL-1β and IL-18 [11, 17]; (2) facilitating non-classical secretory pathway [24]; and (3) causing inflammatory cell death (pyroptosis) or pyrop-apoptosis with plasma membrane rupture, which is in striking contrast to the apoptosis with the features of membrane blebbing and no inflammation [17]. If this was the case, then all the caspase-1 substrates, caspase-1 itself, and inflammasome components should not be localized extracellularly. However, several recent reports challenged this working model [39, 40].

To re-visit this issue, we examined whether caspase-1 substrates, caspase-1, and inflammasome components are localized extracellularly and whether extracellular substrates of caspase-1 can be found in exosomes by searching the UniProKB database. As shown in Table 5, 19 out of 23 caspase-1 substrates localized extracellularly are found in the exosomes, where 8 of these substrates were experimentally verified.

**Table 5:** 19/23 caspase-1 substrates are secreted in exosomes, where 8 of them were experimentally verified

Substrate	Location summary	Extracellular exosome		Inflammation	
		Source^a^	PMID	Inflammatory function	PMID
CA2	Secreted	UniProtKB		Pro-inflammation	7722336
RNH1	Secreted	UniProtKB	19056867		
IL18	Secreted	UniProtKB	23376485	Pro-inflammation	11203186
LDHB	Secreted	UniProtKB			
TBC1D15	Secreted	UniProtKB	23376485		
IL1B	Secreted	UniProtKB		Pro-inflammation	10380697
FAA4	Secreted	UniProtKB			
NUCB2	Secreted	UniProtKB		Pro-inflammation	16407280
SYAP1	Secreted	UniProtKB	18570454		
BID	Secreted	UniProtKB		Pro-inflammation	17209037
AK2	Secreted	UniProtKB	20458337		
TPI1	Secreted	UniProtKB	19056867		
PPP1CA	Secreted				
ENO1	Secreted	UniProtKB		Pro-inflammation	19898480
PSMA7	Secreted	UniProtKB			
HUWE1	Secreted	UniProtKB	19056867		
IL33	Secreted	UniProtKB		Pro-inflammation	18802081
CAT	Secreted	UniProtKB			
CAP1	Secreted	UniProtKB	19056867	Pro-inflammation	24606903
ST14	Secreted	UniProtKB			
IL37	Secreted			Pro-inflammation	22047735
ST14	Secreted				
NEDD4	Secreted				

*Abbreviation*: *PMID* PubMed identifier

^a^UniProtKB/Swiss-Prot location database

Therefore, this raises the question whether pro-caspase-1 gets activated extracellularly in inflammasomes localized in extracellular vesicles. To explore this possibility, a recent report found that microbes or danger signals trigger inflammasome sensors, which induce polymerization of the adapter ASC leading to assembly of an ASC speck. The ASC specks can recruit and activate caspase-1 and induce IL-1β and IL-18 cytokine maturations that result in pyroptotic cell death [39]. After pyroptosis, ASC specks are accumulated in the extracellular space, which further promotes IL-1β cytokine maturation [39], although the finding was disputed by others that ASC complex without NLRP3 is unable to get caspase-1 activated [41]. Along the same line, another report showed that upon activation of caspase-1, oligomeric NLRP3 inflammasome particles were released from macrophages. Recombinant oligomeric protein particles composed of the adaptor ASC or the p.D303N mutant form of NLRP3 associated with cryopyrin-associated periodic syndromes (CAPS) stimulate further activation of caspase-1 extracellularly, as well as intracellularly after phagocytosis by surrounding macrophages [40]. Moreover, caspase-1 is found to be activated in exosomes [42], which can carry caspase-1, cross the injured blood-spinal cord barrier, and deliver the cargo in vivo. Taken together, these analyses suggest that pro-caspase-1 can get activated in extracellular space and that caspase-1 cleaves its substrates in exocytotic secretory pathways including exosomes to propagate inflammation to neighboring and remote cells.

### Most of caspase-1 substrates are upregulated in coronary artery disease (CAD) regardless of their subcellular localization, but different metabolic diseases cause no significant expression changes in caspase-1 nuclear substrates

We hypothesized that if caspase-1 trafficking plays a role in metabolic and autoimmune diseases, the expression and the subcellular localization of caspase-1 substrates may be altered due to changes in the caspase-1 activity. To test this hypothesis, we analyzed RNA transcript expression of the caspase-1 substrates in the microarray datasets deposited in the NIH-NCBI-GEO Datasets database [43].

We examined the expression of caspase-1 substrates in eight disease conditions including coronary artery disease (CAD), metabolic syndrome (MS), type 2 diabetes (T2D), morbidly obese (MO), rheumatoid arthritis (RA), and hypertension. GSE9490 microarray was conducted in vitro on aortic smooth muscle cells in the presence of low and high homocystein (10 μM homocysteine (Hcy) and 100 μM Hcy). As shown in Table 6, we made the following findings: (1) 65 out of 114 (56.5%) caspase-1 substrates are upregulated in coronary heart disease regardless of their subcellular localization, suggesting that caspase-1 and its substrates play a significant role in the pathogenesis of the disease; (2) the majority of metabolic diseases including metabolic syndrome (MS), type 2 diabetes (T2D), morbidly obese (MO), hypertension, and homocystein treatment on aortic smooth muscle cells did not cause significant caspase-1 substrate expression changes, 6.1% (MS), 10.4% (T2D), 5.2% (MO), 5.2% (hypertension), and 6.1% (homocystein treatment on aortic smooth muscle cells); (3) metabolic diseases including metabolic syndrome, type 2 diabetes, morbidly obese, hypertension, and homocysteinemia have no significant expression changes in caspase-1 nuclear substrates; and (4) the 5 caspase-1 substrate that are upregulated in rheumatoid arthritis may play a significant role in the pathogenesis of rheumatoid arthritis.

**Table 6:**
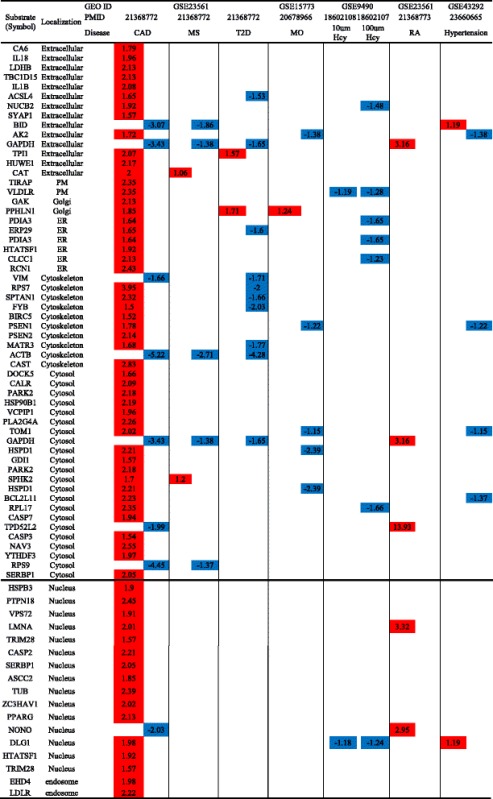
Unlike in metabolic diseases, caspase-1 substrates are upregulated in CAD regardless of their subcellular localization

The numbers in the cells represent the fold change with significance. The colors show the regulatory level (red means upregulation and blue means downregulation)

*Abbreviation*: *CAD*, coronary artery disease, *MS* metabolic syndrome, *T2D* type 2 diabetes, *MO* morbidly obese, *RA* rheumatoid arthritis, *GEO* Gene Expression Omnibus database, *PMID* PubMed identifier

In addition, we examined whether the expression of 38 caspase-1 interaction proteins was changed in these pathological conditions. As shown in Table 7, the expression of 6 out of 38 caspase-1 interaction proteins were changed in metabolic diseases; and 3 and 4 interaction proteins were upregulated in coronary heart disease and hypertension, respectively. These findings suggest that the expression changes of caspase-1 interaction proteins are not a major regulatory mechanism for the pathogenesis of the aforementioned diseases.

**Table 7:**
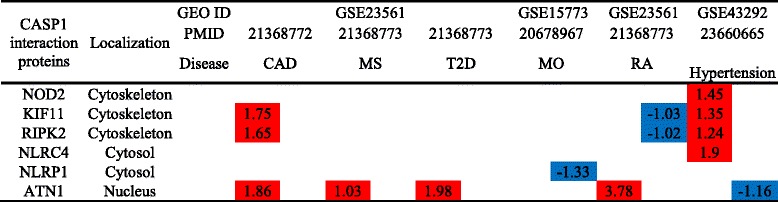
Changes in caspase-1 interaction proteins are insignificant in metabolic and inflammatory diseases

The numbers in the cells represent the fold change with significance. The colors show the regulatory level (red means upregulation and blue means downregulation)

*Abbreviation*: *CASP1* caspase-1, *ID* identification, *CAD* coronary artery disease, *MS* metabolic syndrome, *T2D* type 2 diabetes, *MO* morbidly obese, *RA* rheumatoid arthritis, *GEO* Gene Expression Omnibus database, *PMID* PubMed identifier

Furthermore, to strengthen our argument that most of caspase-1 substrates that are upregulated in coronary heart disease may have pro-inflammatory functions, we examined the expression changes of those caspase-1 substrates in regulatory T cells (Tregs) in comparison to those in T effector cells in five microarray datasets. As shown in Table 8, analyzing of four microarray datasets revealed [44] that the ratios of downregulated caspase-1 substrates versus upregulated substrates were 5/2, 3/3, and 8/4, suggesting that the caspase-1 substrates downregulated in anti-inflammatory/immune suppressive Tregs [45] may be pro-inflammatory in the pathogenesis of coronary heart disease.

**Table 8:**
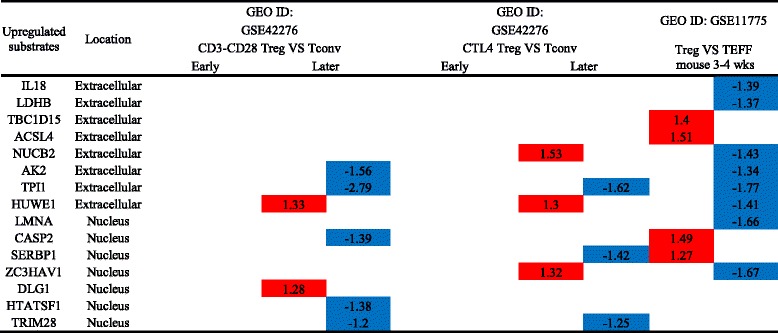
Upregulated caspase-1 extracellular and nuclear substrates in CAD are downregulated in anti-inflammatory T cells

The numbers in the cells represent the fold change with significance. The colors show the regulatory level (red means upregulation and blue means downregulation)

*Abbreviation*: *ID* identification, *KO* knock out, *Treg* regulation T cell, *T* conventional T cell, *GEO* Gene Expression Omnibus database, *PMID* PubMed identifier

### In coronary heart disease, upregulated caspase-1 extracellular substrates are related to inflammatory pathways while caspase-1 nuclear substrates are more related to pathways involved in cell death and chromatin regulation

**Fig. 3:**
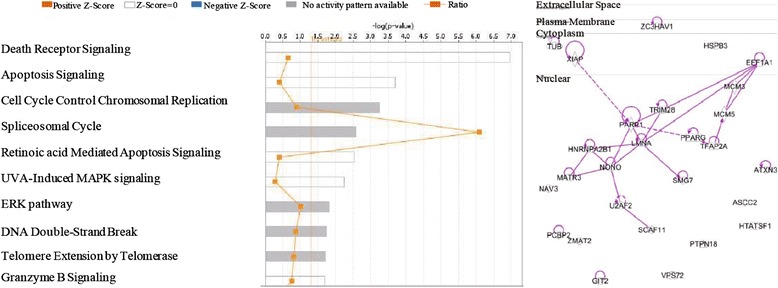
Upregulated caspase-1 nuclear substrates in coronary heart disease induce cell death, chromatin replication, and inflammation

In order to determine the changes of caspase-1 activities in response to the pathogenesis of coronary artery disease, we used the Ingenuity Pathway Analysis (IPA) database as we reported previously [14]. The IPA database is one of the most comprehensive omics data analyzing databases [46] available today; we examined the signaling pathways related to extracellular, cytosolic, and nuclear caspase-1 substrates that were upregulated in coronary artery disease. As shown in Fig. 3, the top ten pathways identified from our Ingenuity Pathway Analysis for upregulated caspase-1 nuclear substrates in coronary artery disease were death receptor signaling, apoptosis signaling, cell cycle control of chromosomal replication, splicesomal cycle, retinoic acid apoptosis signaling, ultraviolet radiation (UVA)-induced mitogen-activated protein kinase (MAPK) signaling, MAPK-extracellular signal-regulated kinase (ERK) pathway, double-strand DNA break repair, telomere extension, and granzyme B signaling. Moreover, as shown in Table 4, three out of seven caspase-1 interaction proteins including androgen receptor (AR), atrophin-1 (ATN1), and CCAAT/enhancer-binding protein beta (CEBPB) have transcription regulatory functions. It is unknown whether caspase-1 interacts with these proteins and fulfills non-catalytic functions in regulating gene transcription in the nucleus.

**Fig. 4:**
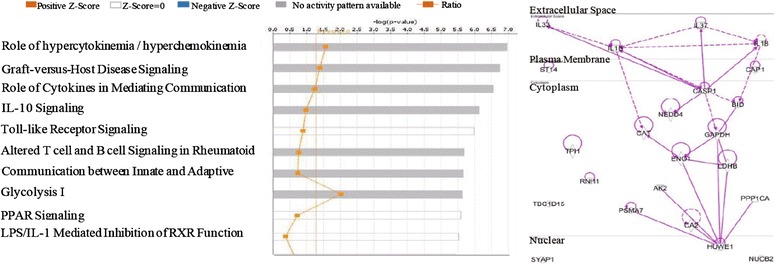
In coronary heart disease, upregulated caspase-1 extracellular substrate-related pathways mediate inflammation

Furthermore, as shown in Fig. 4, we found that the top ten pathways that are related to upregulated caspase-1 extracellular substrates in coronary artery disease are involved in inflammatory signaling including cytokine chemokine secretions, immune responses (graft-versus-host disease), cytokine signaling, and Toll-like receptor signaling. Taken together, these analyses suggest that in coronary artery disease, upregulated caspase-1 extracellular substrates are related to inflammatory pathways while upregulated caspase-1 nuclear substrates are more related to pathways that regulate cell death and chromatin regulation. Surprisingly, most of the cytosolic substrates upregulated during coronary artery disease are significantly involved in ER (endoplasmic reticulum) stress signaling pathway (Additional file 1: Figure S1). Also, our IPA implicated that these upregulated cytosolic substrates may significantly regulate apoptosis, TNF receptor 1 signaling, unfolded protein response, and also ubiquitination.

## Discussion

Recent progress has clearly demonstrated that caspase-1/inflammasome pathway plays a critical role in regulating innate immune system by sensing PAMPs and DAMPs, inducing pro-inflammatory cytokine maturation, promoting multi-format cell death including inflammatory cell death (pyroptosis), pyronecrosis [47], and pyrop-apoptosis, inducing histone modification and unconventional secretion, inhibiting glycolysis, and regulating cell survival [17, 48], eicosanoid storm, autophagy, and metabolism [49]. In addition, myocardial-specific overexpression of caspase-1 induces a significant increase in cardiomyocyte death in young mice without any increase in tissue or plasma levels of IL-1β or other inflammatory mediators [50]. How caspase-1 regulates such broad biological functions is yet unknown. The current working model indicates that pro-caspase-1 gets activated in a protein complex termed inflammasome in the cytosol. However, if the current model is valid, all the caspase-1 substrates should be all localized in the cytosol, which has never been comprehensively examined and verified.

In this study, we examined an important issue whether caspase-1 traffics between intracellular organelles for cleaving its substrates. To investigate this hypothesis, we took novel proteomic data analysis, protein interaction data, and protein intracellular localization database approaches, and analyzed intracellular localization of 114 experimentally identified caspase-1 substrates and 38 caspase-1 interaction proteins. Our analysis revealed the following important findings:

1. *Caspase-1 substrates and interaction proteins are localized in various intracellular organelles*. Our data reveals the presence of caspase-1 substrates and interaction proteins in variety of subcellular compartments, indicating the activity of caspase-1 may extend beyond the cytosol. There are experimental evidence confirming the formation of active inflammasomes in the nucleus and in extracellular vesicles, which we have elaborated below. We also found the presence of caspase-1 substrates and interaction proteins in Golgi bodies, ER, and mitochondria. So far, formation of active inflammasomes and active caspase-1 in these three compartments are not experimentally verified. Our data suggests that caspase-1 may play an active role in these organelles that may contribute to inflammatory disease progression, therefore emphasizing the need for experimental verification of its role extensively in subcellular compartment other than the cytosol.
2. *Caspase-1 gets activated in situ in the nucleus in response to intra-nuclear danger signals.* Previously, pro-caspase-1 [22] and inflammatory components such as NLRP3 [51] and ASC (apoptosis-associated speck-like protein containing a carboxy-terminal CARD) [34] were found to shuttle between nucleus and cytoplasm in response to various inflammatory responses. Interestingly, formation of an active inflammasome in the nucleus was reported during viral infections. For an example, *Kerur et al*. clearly demonstrated that pro-caspase-1, ASC, and IFI16 make active nuclear inflammasomes that lead to production of activated caspase-1 in the nucleus in the presence of Kaposi sarcoma virus (KSHV) infection in dermal endothelial cells [35]. Also, the same group had shown that inflammasomes comprised of IFI16, ASC, and pro-caspase-1 are formed in the nucleus in response to Epstein-Barr virus (EBV) infections as well [52]. However, unlike the inflammasomes that were formed due to KSHV infection, inflammasomes that are made in response to EBV infection seem to produce active caspase-1 only in the cytoplasm. Nevertheless, based on our findings, there is a possibility that these inflammasomes constructed due to viral infections may trigger cellular signaling pathways in the nucleus that are yet to be explored. Even though formation of an active nuclear inflammasome is not demonstrated, *Aries et al.* implicated the presence of active caspase-1 in the nucleus that cleaves the transcription factor GATA4, which negatively impacts the cell survival of cardiomyocytes treated with doxorubicin [19]. Furthermore, we also have demonstrated that pro-atherogenic stimuli-mediated induction of active caspase-1 cleaves Sirt-1, a histone deacetylase predominantly found in the nucleus [11]. How the active caspase-1 and inflammasome components shuttle in and out of the nucleus is not clearly understood. However, presence of nuclear localization signal (NLS) in the N-terminal of pro-caspase-1 was previously reported [22]. Nonetheless, whether caspase-1 and other inflammatory components in the nucleus play a significant role during the progression of inflammatory disorders other than during the viral infections and pharmacological interventions such as doxorubicin have not been studied. The nuclear danger signals that are documented to prompt in situ activation of caspase-1 or inflammasome formation are summarized in Additional file 1: Table S4. The data provided in the current manuscript suggest the possibility of formation of a variety of active inflammasome complexes in the nucleus in response to various stimuli. Also it provides potential nuclear caspase-1 substrates that need experimental validation in the future.
3. *Caspase-1 cleaves its substrates on exocytotic secretory pathways including exosomes to propagate inflammation to neighboring and remote cells.* Previous reports have provided evidence that activated caspase-1 is localized in microvesicles shed by monocytes in response to inflammation [53] and also that caspase-1 is secreted extracellularly by Golgi/ER-independent manner [24]. Moreover, it was also shown that pro-caspase-1 gets activated extracellularly and processes and induces the secretion of mature IL-1β from platelet microparticles [54], which propagates inflammation in a paracrine manner [40]. Nevertheless, whether or not pro-caspase-1 and other proteins that form the inflammasomes are involved in production of active caspase-1 in membrane vesicles shed by cells is not fully understood. Our analysis revealed the possibility of formation of active inflammasome complexes in these vesicles, which may eventually propagate inflammation via active caspase-1 under inflammatory stimuli and pathological conditions.
4. *Caspase-1 substrates are upregulated in coronary artery disease.* Previously, we demonstrated that pro-caspase-1 and its active p20 subunit expression was significantly upregulated in aortas of ApoE-deficient mice during early hyperlipidemia (high-fat diet feeding for 3 weeks) [11]. Moreover, we demonstrated that caspase-1 deficiency in ApoE^−/−^ background significantly attenuated the atherosclerotic lesion formation in aortic sinus of the mice fed with high-fat diet for 3 weeks [11]. Interestingly, in the current study, we found that most of caspase-1 substrates are upregulated in coronary artery disease regardless of their subcellular localization. Therefore, our findings suggest that caspase-1 and its substrates may play a crucial role during the disease progression of coronary artery disease. Also, a previous publication had demonstrated increased expression of caspase-1 and NLRP3 inflammasome in the adipose tissue and liver of obese mice and humans [55]. Furthermore, the same study implicated that the level of caspase-1 expression was positively correlated with the severity of type 2 diabetes in individuals. However, in the current study, we could not observe any significant changes in the expression of caspase-1 substrates in different cellular compartments in metabolic diseases.
5. *Upregulated caspase-1 substrates in different compartments trigger differential signaling pathways.* In coronary artery disease, majority of upregulated caspase-1 extracellular substrates are components of inflammatory signaling pathways; and in contrast, most of the upregulated caspase-1 nuclear substrates are involved in cell death and chromatin regulation. Also, our analysis revealed that the upregulated cytosolic caspase-1 substrates in coronary artery disease regulate ER stress (Additional file 1: Figure S1). Previous publications have confirmed that ER stress can be a potential intracellular danger signal for caspase-1 activation (Additional file 1: Table S4). Thus, our data suggests potential downstream targets of caspase-1 that may play a significant role in inflammatory disorders such as coronary artery disease. Further studying of these substrates and interaction proteins may reveal probable targets that can be therapeutically intervened for treatment of chronic inflammatory disorders in the future.

Our analysis revealed potential novel substrates and interaction proteins of caspase-1 that may exert biological effects in various cellular compartments and induce diverse reactions. However, recent work also provides the evidence that the biological effects exerted by caspase-1 do not solely depend on its protease activity. In contrast to its usual inhibitory effects on proteins by proteolytic cleavage, caspase-1 was found to activate caspase-7 [56] and also increase the activity of NF-kB independent of its enzymatic activity [57]. Furthermore, caspase-1 activity can be regulated by post-translational modifications such as deubiquitination and glutathionylation [58, 59]. Therefore, this suggest that the function of caspase-1 is far more intricate and it may interact with some of its substrates and interactions proteins via non-canonical pathways, which are yet to be elucidated.

We conducted a comprehensive analysis on raw data available in databases that contained information that were already experimentally validated, and hence, our work deviates from a traditional literature review where a summary is presented on the already existing literature. All the publications that we have cited in this article is to further strengthen and validate the novel working model of caspase-1 we proposed. Furthermore, the models we suggest here were based on experimentally generated data but not on bioinformatics predictions although bioinformatics approaches have significantly improved our understanding on many complex biological issues [60]. All the caspase-1 substrates analyzed in this study were reported previously [27, 28] and were identified using mass-spectrometry-based proteomic approaches. To enhance the strengths of the data, we verified the reliability of two different subcellular localization databases [31] of proteins [30] by analyzing 21 well-characterized subcellular organelle protein markers. In analyzing the caspase-1 substrate expression changes, we analyzed the microarray experimental data sets deposited in the NIH-GEO Datasets database.

One of the limitations in this manuscript is that the data presented relies mainly on microarray data, which depicts gene expression changes but may not necessarily reflect the protein expression. Furthermore, we acknowledge that the list of caspase-1 substrates and interact proteins were obtained only from in vitro experiments that intracellular compartmental structures are not well maintained. However, most of the caspase-1 substrates and the interaction proteins identified in this study seem to exist in cellular compartments other than the cytosol. Therefore, further studies should be conducted to determine the pathophysiological relevance of caspase-1 cleavage of the identified substrates and caspase-1 binding to no-substrate interaction proteins in vivo and also in clinical settings.

## Conclusions

**Fig. 5:**
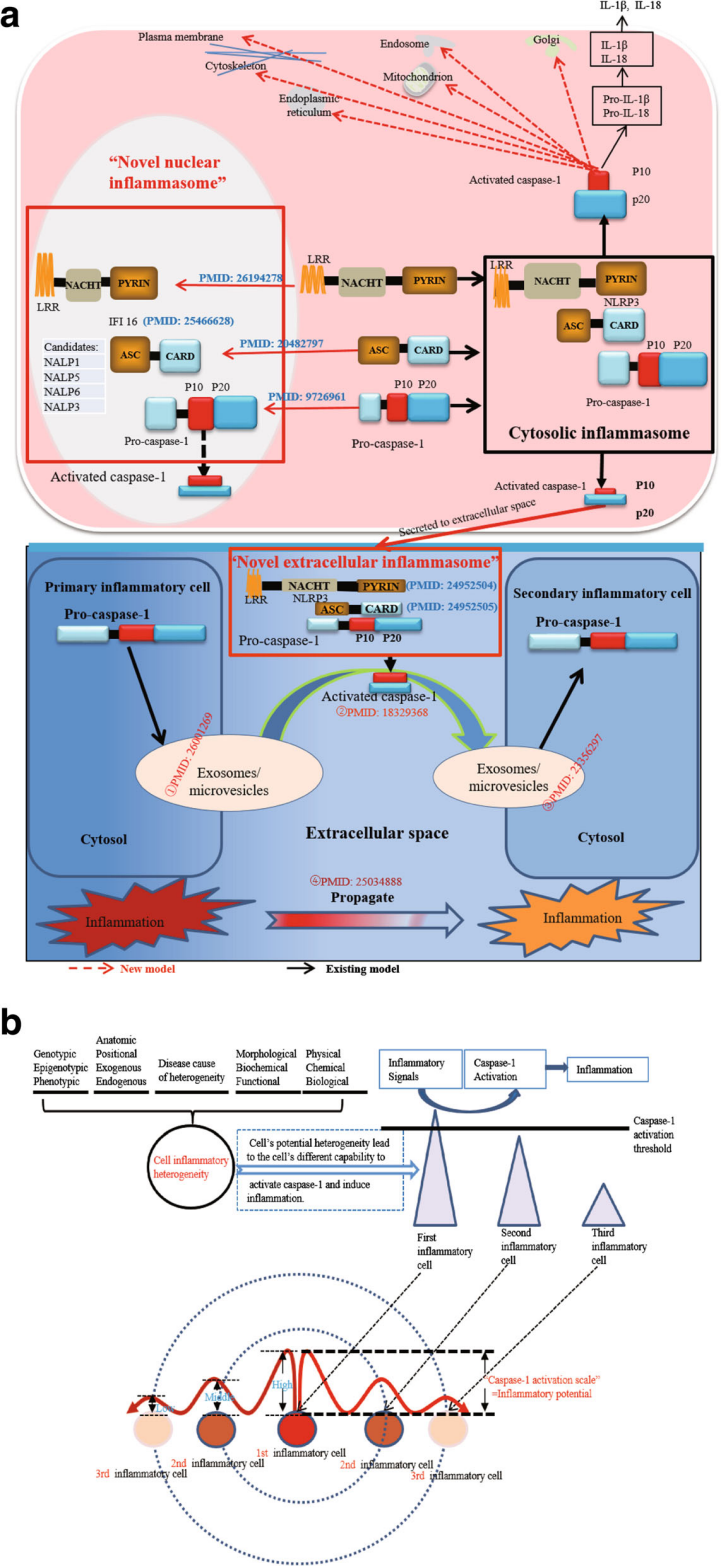
Proposed novel caspase-1 working models. **a** Experimentally identified substrates and interaction proteins indicate that caspase-1 may traffic to subcellular locations other than the cytosol. Caspase-1 gets activated in situ in the nucleus by novel nuclear inflammasomes and caspase-1 cleaves its substrates in exocytotic secretory pathways including exosomes to propagate inflammation to neighboring and remote cells. *A-CASP1:* activated caspase-1. ① The release of extracellular vesicles (Evs) as a mode of intercellular communication. ② Caspase-1 by inflammasome complexes is directly linked to exosomes and microvesicles (EMVs) that acts as a transport vehicle in this pathway. ③ Exosomes and microvesicles (EMVs) can transmit signals and molecules to neighboring cells via a non-viral pathway of intercellular vesicle traffic. ④ Breast cancer cells induce pro-inflammatory activity of distant macrophages through circulating exosomal vesicles secreted during cancer progression—so-called cancer propagation. **b** Novel model of extracellular trafficking of caspase-1 and inflammasome components propagate inflammation to neighboring and remote cells

Our new results and others’ recent papers allow us to propose a new working model illustrated in Fig. 5a: *first*, in response to the stimulation of conditional and classical DAMPs [61] and PAMPs, caspase-1 can be activated in inflammasome complexes in cytosol, nucleus and extracellularly; *second*, several types of novel nuclear inflammasomes can be assembled and get pro-caspase-1 activated in situ in the nucleus in response to intra-nuclear danger signals related to cell death and chromatin regulation; *third*, cytosolic inflammasomes and novel extracellular inflammasomes are more functional in promoting inflammation in response to extracellular and cytosolic danger signals; *fourth*, due to various factors listed in Fig. 5b, cells are heterogeneous in the speed in activating caspase-1 and initiating inflammation in responding to extracellular and cytosolic danger signals, which we coined the term as inflammation privilege in 2009 [8]. The cells respond to danger signals first will propagate the inflammation via sending out activated caspase-1-based exosomes and ASC specks, which can be uptake by neighboring and remote cells that are relatively slower in sensing danger signals and make those cells inflamed. Our identification of novel caspase-1 trafficking organelles, nuclear inflammasomes, extracellular inflammasomes and extracellular caspase-1-based inflammation propagation model significantly improves our understanding of caspase-1 function and provides a list of targets for the future development of new therapeutics to treat cardiovascular diseases, inflammatory diseases, and inflammatory cancers.

## Additional file

Additional file 1: Table S1. Analysis of 21 experimentally verified subcellular markers to confirm the reliability of two different databases. Table S2. 114 experimentally identified caspase-1 substrates are localized in various organelles including nucleus and secreted extracellularly. Table S3. 38 experimentally verified caspase-1 interaction proteins are localized in various intracellular organelles. Table S4. Danger signals involved in inflammasome activation. Figure S1. In coronary artery disease, the signal pathway of caspase-1 upregulated cytosolic, nuclear and extracellular substrates. (DOCX 828 kb)
