# Supplementary material for: Novel extracellular and nuclear caspase-1 and inflammasomes propagate inflammation and regulate gene expression: a comprehensive database mining study
Source: J Hematol Oncol. 2016 Nov 14;9:122. doi: 10.1186/s13045-016-0351-5 (PMC5109738; doi:10.1186/s13045-016-0351-5)

Additional file 1

Table S1. Analysis of 21 experimentally verified subcellular markers to confirm the reliability of two different databases.

| Location markers (Full name) | Symbol | PMID | Location  (Verified) | Subcellular locations | |
| --- | --- | --- | --- | --- | --- |
|  |  |  |  | Database 1 (Confidence)# | Database 2& |
| ATPase Na+/K+ transporting subunit alpha 1 | ATP1A1 | 1975705 | PM | PM (5) | Cell membrane |
| ATPase plasma membrane Ca2+ transporting 1 | ATP2B1 | 8396145 |  | PM (5) | Cell membrane |
| Heat shock protein 90kDa alpha family class A member 1 | HSP90AA1 | 12526792 | cytosol | Cytosol (5) | Cytoplasm |
| Glyceraldehyde-3-phosphate dehydrogenase | GAPDH | 26740924 |  | Cytosol (4) | cytosol |
| Caveolin-1 | CAV1 | 20682791 | Endosome | Endosome (5) | Peripheral membrane |
| Early endosome antigen 1 | EEA1 | 11741531 |  | Endosome (5) | Early endosome membrane |
| Protein disulfide isomerase family A member 4 | PDIA4 | 26170458 | ER | ER (5) | ER lumen |
| Prolyl 4-hydroxylase subunit beta | P4HB | 12095988 |  | ER (5) | ER lumen |
| Calreticulin | CALR | 16130169 |  | ER (5) | ER lumen |
| Golgin A2 | GOLGA2 | 15229288 | Golgi | Golgi apparatus (5) | Golgi apparatus |
| Estrogen receptor binding site associated, antigen, 9 | EBAG9 | 10426319 |  | Golgi apparatus (4) | Golgi apparatus membrane |
| Golgin A1 | GOLGA1 | 17724343 |  | Golgi apparatus (4) | Golgi apparatus |
| Apoptosis inducing factor, mitochondria associated 1 | AIFM1 | 20833797 | Mito | Mito (5) | Mito intermembrane space |
| Cytochrome c oxidase subunit 4I2 | COX4I2 | 11311561 |  | Mito (5) | Mito inner membrane. |
| Cytochrome c | CYCS | 20671748 |  | Mito (5) | Mito intermembrane space |
| Beta-actin | ACTB | 16130169 | cytoskeleton | Cytoskeleton (5) | Cytoskeleton |
| Vimentin | VIM | 16130169 |  | Cytoskeleton (5) | Cytoplasm |
| Keratin 18 | KRT18 | 26126491 |  | Cytoskeleton (5) | Cytoplasm |
| Sp1 transcription factor | SP1 | 3139301 | Nuclear | Nucleus (5) | Nucleus |
| Histone deacetylase 2 | HDAC2 | 18347167 |  | Nucleus (5) | Nucleus |
| Histone deacetylase 3 | HDAC3 | 12711221 |  | Nucleus (5) | Nucleus |

Abbreviation: PM: plasma membrane; ER: endoplasmic reticulum; Mito: mitochondrion; #: Compartments subcellular location database; &: UniProtKB/Swiss-Prot location database(<http://www.genecards.org/>); PMID: PubMed identifier; Confidence range from 1-5, where 5 indicates the maximum confidence.

Table S2. 114 experimentally identified caspase-1 substrates are localized in various organelles including nucleus and secreted extracellularly.

| Substrate (Full name) | Symbol | Source | Subcellular localization (probabilities) | | | | |
| --- | --- | --- | --- | --- | --- | --- | --- |
|  |  |  | Database 1 # | | | Database 2 & | Summary* |
|  |  |  | High | Middle | Low |  |  |
| Carbonic anhydrase 2 | CA2 | 2 | Extracellular | PM | Cytosol | Secreted | Secreted |
| Ribonuclease inhibitor | RNH1 | 2 | Extracellular | Cytosol | Chloroplast | Cytoplasm | Secreted |
| Pro-IL-18 | IL18 | 1 | Extracellular | Cytosol | Golgi | Secreted | Secreted |
| L-lactate dehydrogenase B chain | LDHB | 2 | Extracellular | Cytosol | Mito | Cytoplasm | Secreted |
| TBC1 domain family member 15 | TBC1D15 | 2 | Extracellular | Cytosol | Mito | Cytoplasm | Secreted |
| Pro-IL-1β | IL1B | 2 | Extracellular | Cytosol | Nucleus | Secreted | Secreted |
| Long-chain-fatty-acid--CoA ligase 4 | FAA4 | 2 | Extracellular | Cytosol | Cytoskeleton | Melanosome | Secreted |
| Nucleobindin-2 | NUCB2 | 1 | Extracellular | Cytosol | Golgi | Secreted | Secreted |
| Synapse-associated protein 1 | SYAP1 | 2 | Extracellular | Golgi | Nucleus |  | Secreted |
| BH3-interacting domain death agonist | BID | 2 | Extracellular | Mito | Cytosol | Cytoplasm | Secreted |
| Adenylate kinase-2 | AK2 | 2 | Extracellular | Mito | Cytosol | Mito | Secreted |
| Triosephosphate isomerase | TPI1 | 2 | Extracellular | Nucleus | Cytosol |  | Secreted |
| Protein phosphatase 1, catalytic subunit, alpha | PPP1CA | 2 | Extracellular | Nucleus | Cytosol | Cytoplasm | Secreted |
| α-enolase | ENO1 | 2 | Extracellular | Nucleus | Cytosol | Cytoplasm | Secreted |
| Proteasome subunit alpha type-7 | PSMA7 | 2 | Extracellular | Nucleus | Cytosol | Cytoplasm | Secreted |
| E3 ubiquitin-protein ligase HUWE1 | HUWE1 | 2 | Extracellular | Nucleus | Cytosol | Cytoplasm | Secreted |
| Interleukin 33 | IL33 | 2 | Extracellular | Nucleus | Cytosol | Secreted | Secreted |
| Catalase | CAT | 2 | Extracellular | peroxisome | Cytosol | Peroxisome | Secreted |
| Adenylyl cyclase-associated protein 1 | CAP1 | 2 | Extracellular | PM | Cytoskeleton | Cell membrane | Secreted |
| Suppressor of tumorigenicity 14 protein | ST14 | 2 | Extracellular | PM |  | Membrane | Secreted |
| Interleukin 37 | IL37 | 1 | Extracellular | Cytosol | Nucleus | Secreted | Secreted |
| Nedd4 | NEDD4 | 1 | Extracellular | PM | Golgi | Cytoplasm | Secreted |
| Putative maltase-glucoamylase-like protein | MGAM2 | 2 | PM |  |  | Membrane | PM |
| Toll/interleukin-1 receptor domain-containing adapter protein | TIRAP | 2 | PM | Cytosol | Extracellular | Membrane | PM |
| LIM domain-containing protein 1 | LIMS1 | 1 | PM | Cytosol |  | Membrane | PM |
| Very low-density lipoprotein receptor | VLDLR | 2 | PM | Extracellular | Cytoskeleton | Membrane | PM |
| C-C motif chemokine 3 | CCR3 | 1 | PM | Extracellular | Extracellular | Membrane | PM |
| ADP-ribosylation factor GTPase-activating protein 2 | ARFGAP2 | 1 | Golgi | Nucleus | PM | Golgi | Golgi |
| Cyclin G-associated kinase | GAK | 2 | Golgi | lysosome | PM | Golgi | Golgi |
| Periphilin-1 | PPHLN1 | 2 | Golgi | Nucleus | Cytosol | Cytoplasm | Golgi |
| Calumenin | CALU | 1 | ER | Extracellular | Golgi | ER | ER |
| Protein disulfide-isomerase A3 | PDIA3 | 2 | ER | Extracellular | Nucleus | ER | ER |
| Phosphatidylinositol 4-kinase beta | PI4KB | 2 | ER | Golgi | Mito | Cytoplasm | ER |
| Chloride channel CLIC-like protein 1 | CLCC1 | 1 | ER | Golgi | Nucleus | ER | ER |
| Cyclophilin B | CYPB | 2 | ER | Extracellular | Nucleus | ER | ER |
| Reticulocalbin-1 | RCN1 | 1 | ER |  |  | ER | ER |
| Arginine/serine-rich coiled-coil protein 1 | RSRC1 | 2 | ER | Golgi | Mito | Mito | ER |
| Malate dehydrogenase | MDH | 1 | Mito | peroxisome | Cytosol | Mito | Mito |
| Vimentin | VIM | 1 | Cytoskeleton | Cytosol | Extracellular | Cytoskeleton | Cytoskeleton |
| MAP-Tau Isoform 2 | MAPT | 1 | Cytoskeleton | Nucleus | PM | Cytoskeleton | Cytoskeleton |
| SPTAN1 | SPTAN1 | 1 | Cytoskeleton | Extracellular | Cytosol | Cytoskeleton | Cytoskeleton |
| FYN-binding protein | FYB | 2 | Cytoskeleton | Cytosol | Nucleus | Cytoplasm | Cytoskeleton |
| PSEN1 | PSEN1 | 1 | Cytoskeleton | ER | Golgi | ER | Cytoskeleton |
| PSEN2 | PSEN2 | 1 | Cytoskeleton | Nucleus | ER | Membrane | Cytoskeleton |
| Actin ,cytoplasmic 1 | ACTG1 | 1 | Cytoskeleton | Extracellular | Nucleus | Cytoskeleton | Cytoskeleton |
| Rho GDP-dissociation inhibitor 2 | ARHGDIB | 2 | Cytoskeleton | Extracellular | Cytosol | Cytoplasm | Cytoskeleton |
| F-actin capping protein alpha-1 | CAPZA1 | 2 | Cytoskeleton | Extracellular | Cytosol | Cytoskeleton | Cytoskeleton |
| β-Actin | ACTB | 1 | Cytoskeleton | Extracellular | Nucleus | Cytoskeleton | Cytoskeleton |
| γ-Actin | ACTG1 | 2 | Cytoskeleton | Extracellular | Nucleus | Cytoskeleton | Cytoskeleton |
| Nucleoprotein TPR | TPR | 1 | Cytoskeleton | Nucleus | Extracellular | Cytoskeleton | Cytoskeleton |
| Heterogeneous nuclear ribonucleoprotein K | HNRNPK | 1 | Cytoskeleton | Cytosol |  | Cytoplasm | Cytoskeleton |
| Zyxin | ZYX | 1 | Cytoskeleton | Nucleus | PM | Cytoskeleton | Cytoskeleton |

Abbreviation: 1: From published paper(PMID:20173201); 2: From published paper(PMID:22764097); PM: plasma membrane; ER: endoplasmic reticulum; Mito: mitochondrion; #: Compartments subcellular location database; &: UniProtKB/Swiss-Prot location database; *: Summary data to see figure 2, and the red is confirmed by both database 1 and database 2.

Table S2. 114 experimentally identified caspase-1 substrates are localized in various organelles including nucleus and secreted extracellularly(continue).

| Substrate ( Full name) | Symbol | Source | Subcellular localization (probabilities) | | | | |
| --- | --- | --- | --- | --- | --- | --- | --- |
|  |  |  | Database 1 # | | | Database 2 & | Summary |
|  |  |  | High | Middle | Low |  |  |
| Dedicator of cytokinesis protein 5 | DOCK5 | 1 | Cytosol | Cytoskeleton |  | Cytoplasm | Cytosol |
| Glyceraldehyde-3-phosphate dehydrogenase | GAPDH | 2 | Cytosol | Cytoskeleton | Nucleus | Cytosol | Cytosol |
| Rap1-GTP-interacting adapter molecule | APBB1IP | 2 | Cytosol | Cytoskeleton | PM | Cytoplasm | Cytosol |
| Sequestosome-1 | SQSTM1 | 1 | Cytosol | ER | endosome | Cytoplasm | Cytosol |
| Calreticulin | CALR | 2 | Cytosol | ER | Extracellular | Cytosol | Cytosol |
| Parkin | PARK2 | 1 | Cytosol | Mito | Golgi | Cytosol | Cytosol |
| Endoplasmin | HSP90B1 | 1 | Cytosol | ER | Extracellular | ER | Cytosol |
| Cytosolic phospholipase A2 | PLA2G4A | 2 | Cytosol | ER | Mito | Cytoplasm | Cytosol |
| Protein SET | SET | 2 | Cytosol | ER | Nucleus | Cytosol | Cytosol |
| Target of Myb protein 1 | TOM1 | 2 | Cytosol | Endosome | Extracellular | Cytoplasm | Cytosol |
| Hsp60 | HSPD1 | 2 | Cytosol | Extracellular | Mito | Mito | Cytosol |
| Ras-related C3 botulinum toxin substrate 2 | RAC2 | 2 | Cytosol | Extracellular | PM | Cytoplasm | Cytosol |
| Rab GDP dissociation inhibitor alpha | GDI1 | 2 | Cytosol | Golgi | Cytoskeleton | Cytoplasm | Cytosol |
| Pyrin | NLRP3 | 1 | Cytosol | Extracellular | lysosome | Cytoplasm | Cytosol |
| Sphingosine kinase 2 | SPHK2 | 2 | Cytosol | lysosome | vacuole | Cytoplasm | Cytosol |
| 60S ribosomal protein L27 | RPL27 | 1 | Cytosol | Extracellular | Nucleus |  | Cytosol |
| BCL-XL | BCL2L11 | 1 | Cytosol | Mito | Nucleus | Mito | Cytosol |
| 60S ribosomal protein L17 | RPL17 | 1 | Cytosol | Nucleus |  |  | Cytosol |
| Zinc finger CCCH domain-containing protein 4 | ZC3H4 | 2 | Cytosol | Nucleus | Cytoskeleton |  | Cytosol |
| Caspase-7 | CASP7 | 2 | Cytosol | Nucleus | ER | Cytoplasm | Cytosol |
| 60S ribosomal protein L4 | RPL4 | 1 | Cytosol | Nucleus | ER |  | Cytosol |
| Tumor protein D54 | TPD52L2 | 1 | Cytosol |  |  |  | Cytosol |
| Caspase-3 | CASP3 | 2 | Cytosol | Nucleus | Mito | Cytoplasm | Cytosol |
| Gasdermin D | GSDMD | 1 | Cytosol | Nucleus |  |  | Cytosol |
| Eukaryotic translation initiation factor 3 subunit J | EIF3J | 1 | Cytosol |  |  | Cytoplasm | Cytosol |
| Hsp90 | HSP90AA1 | 1 | Cytosol | Extracellular | Nucleus | Cytoplasm | Cytosol |
| Caprin-1 | CAPRIN1 | 1 | Cytosol | PM |  | Cytosol | Cytosol |
| Plasminogen activator inhibitor 1 RNA-binding protein | SERBP1 | 1 | Cytosol | Extracellular | Nucleus | Cytoplasm | Cytosol |
| 40S ribosomal protein S7 | RPS7 | 1 | Cytosol | Extracellular | Nucleus | Cytoplasm | Cytosol |
| Calpastatin | CAST | 1 | Cytosol | Cytoskeleton | ER |  | Cytosol |
| Peroxiredoxin-6 | PRDX6 | 2 | Cytosol | Extracellular | Cytoskeleton | Cytoplasm | Cytosol |
| Plasminogen activator inhibitor 1 RNA-binding protein | SERBP1 | 1 | Cytosol | Extracellular | PM | Cytoplasm | Cytosol |
| Heat shock protein beta-3 | HSPB3 | 2 | Nucleus | Cytoskeleton | Cytosol | Nucleus | Nucleus |
| HNRNPA2B1 | HNRNPA2B1 | 1 | Nucleus | Cytoskeleton |  | Nucleus | Nucleus |
| SMG7 | SMG7 | 2 | Nucleus | Cytoskeleton | Cytosol | Nucleus | Nucleus |
| TF AP-2α | TFAP2A | 1 | Nucleus | Cytoskeleton | Golgi | Nucleus | Nucleus |
| Tyrosine-protein phosphatase non-receptor type 18 | PTPN18 | 2 | Nucleus | Cytoskeleton | Mito | Nucleus | Nucleus |
| DNA replication licensing factor MCM3 | MCM3 | 2 | Nucleus | Cytoskeleton |  | Nucleus | Nucleus |
| Vacuolar protein sorting-associated protein 72 homolog | VPS72 | 2 | Nucleus | Cytoskeleton |  | Nucleus | Nucleus |
| Elongation factor 1-alpha 1 | EEF1A1 | 1 | Nucleus | Cytosol |  | Nucleus | Nucleus |
| LMNA Isoform 2 | LMNA | 1 | Nucleus | Cytosol |  | Nucleus | Nucleus |
| ARF GTPase-activating protein GIT2 | GIT2 | 1 | Nucleus | Cytoskeleton |  |  | Nucleus |
| Poly(rC)-binding protein 2 | PCBP2 | 1 | Nucleus | Cytosol |  | Nucleus | Nucleus |
| PARP | PARP1 | 1 | Nucleus | Cytosol | Mito | Nucleus | Nucleus |
| Ataxin-3 | ATXN3 | 1 | Nucleus | Cytosol | Mito | Nucleus | Nucleus |
| Splicing factor U2AF 65 kDa subunit | U2AF2 | 1 | Nucleus |  |  | Nucleus | Nucleus |
| Caspase-2 | CASP2 | 2 | Nucleus | Cytosol | Mito |  | Nucleus |
| HIV Tat-specific factor 1 | HTATSF1 | 1 | Nucleus |  |  | Nucleus | Nucleus |
| Baculoviral IAP repeat-containing protein 4 | BIRC4 | 2 | Nucleus | Cytosol | Mito | Nucleus | Nucleus |
| Matrin-3 | MATR3 | 1 | Nucleus |  |  | Nucleus | Nucleus |
| Neuron navigator 3 | NAV3 | 1 | Nucleus | Cytosol |  | Nucleus | Nucleus |
| Activating signal cointegrator 1 complex subunit 2 | ASCC2 | 2 | Nucleus | Cytosol |  |  | Nucleus |
| Protein SCAF11 | SCAF11 | 2 | Nucleus | Cytosol |  | Nucleus | Nucleus |
| β-tubulin | TUB | 2 | Nucleus | Extracellular | PM | Nucleus | Nucleus |
| Zinc finger CCCH domain-containing protein | ZC3HAV1 | 1 | Nucleus | Golgi | PM | Nucleus | Nucleus |
| Zinc finger matrin-type protein 2 | ZMAT2 | 1 | Nucleus | Mito |  | Nucleus | Nucleus |
| PPAR-γ | PPARG | 1 | Nucleus | peroxisome | Cytosol | Nucleus | Nucleus |
| Non-POU domain-containing octamer-binding protein | NONO | 1 | Nucleus | Cytosol | Extracellular | Nucleus | Nucleus |
| DNA replication licensing factor MCM5 | MCM5 | 1 | Nucleus |  |  | Nucleus | Nucleus |
| TIF1b | TRIM28 | 2 | Nucleus |  |  | Nucleus | Nucleus |
| Growth factor receptor-bound protein 2 | GRB2 | 2 | Endosome | Extracellular | Nucleus | Endosome | Endosome |
| Very low-density lipoprotein recepor | LDLR | 1 | Endosome | Golgi | lysosome | Endosome | Endosome |

Abbreviation: 1: From published paper(PMID:20173201); 2: From published paper(PMID:22764097); PM: plasma membrane; ER: endoplasmic reticulum; Mito: mitochondrion; #: Compartments subcellular location database; &: UniProtKB/Swiss-Prot location database; *: Summary data to see figure 2, and the red is confirmed by both database 1 and database 2.

Table S3. 38 experimentally verified caspase-1 interaction proteins are localized in various intracellular organelles.

| CASP1 interaction proteins Ѱ | Symbol | PMID | Subcellular localization (probabilities) | | | | |
| --- | --- | --- | --- | --- | --- | --- | --- |
|  |  |  | from database 1 # | | | From database 2 & | Summary* |
|  |  |  | High | Middle | Low |  |  |
| Caspase 14 | CASP14 | 9792675 | Extracellular | Nucleus | Cytoskeleton | Cytoplasm | Secreted |
| Kinesin family member 3A | KIF3A | 26186194 | Extracellular | Cytoskeleton | Cytosol | Secreted | Secreted |
| Epidermal growth factor receptor | EGFR | 11226410 | PM | ER | Extracellular | Membrane | PM |
| Nucleotide binding oligomerization domain containing 1 | NOD1 | 12459189 | PM | Cytosol | Endosome | Membrane | PM |
| P21 (RAC1) activated kinase 1 | PAK1 | 15561713 | PM | Cytosol | Golgi | Membrane | PM |
| Nucleotide binding oligomerization domain containing 2 | NOD2 | 15107016 | PM | Cytoskeleton | Cytosol | Membrane | PM |
| TIR domain containing adaptor protein [Homo sapiens | TIRAP | 20048342 | PM | Cytosol | Extracellular | Membrane | PM |
| X-linked Kx blood group [Homo sapiens | XK | 15107016 | PM |  |  | Membrane | PM |
| Caspase recruitment domain family member 16 | CARD16 | 11536016 | Golgi |  |  |  | Golgi |
| Presenilin 1 | PSEN1 | 10069390 | ER | Cytoskeleton | Golgi | ER | ER |
| B-cell receptor-associated protein 31 | BCAP31 | 9334338 | ER | PM | Cytosol | ER | ER |
| Phospholipase A2 group IVB | PLA2G4B | 9875225 | ER | Golgi | Mito | ER | ER |
| BCL2 like 1 | BCL2L1 | 9435230 | Mito | Cytoskeleton | Nucleus | Mito | Mito |
| BH3 interacting domain death agonist | BID | 11085743 | Mito | Extracellular | Cytosol | Mito | Mito |
| Receptor interacting serine/threonine kinase 2 | RIPK2 | 9705938 | Cytoskeleton | Cytosol | Nucleus | Cytoplasm | Cytoskeleton |
| Kinesin family member 11 | KIF11 | 26186194 | Cytoskeleton | Cytosol | Nucleus | Cytoskeleton | Cytoskeleton |
| NLR family CARD domain containing 4 | NLRC4 | 15107016 | Cytosol | Cytoskeleton | Nucleus | Cytosol | Cytosol |
| NLR family pyrin domain containing 1 | NLRP1 | 17418785 | Cytosol | Nucleus |  | Cytoplasm | Cytosol |
| Huntingtin [Homo sapiens | HTT | 9535906 | Cytosol | ER | Endosome | Cytoplasm | Cytosol |
| Caspase recruitment domain family member 17 | CARD17 | 15383541 | Cytosol | Mito |  | Cytoplasm | Cytosol |
| Caspase recruitment domain family member 8 | CARD8 | 11821383 | Cytosol | Nucleus |  | Cytoplasm | Cytosol |
| Calpastatin | CAST | 9705209 | Cytosol | Cytoskeleton | ER |  | Cytosol |
| Nuclear factor, erythroid 2 like 2 | NFE2L2 | 10510468 | Cytosol | Nucleus | Cytoskeleton | Cytosol | Cytosol |
| PYD and CARD domain containing | PYCARD | 12191486 | Cytosol | ER | Mito | Cytoplasm | Cytosol |
| Caspase 10 | CASP10 | 8962078 | Cytosol | PM | Mito |  | Cytosol |
| Caspase 8 | CASP8 | 8962078 | Cytosol | PM | Cytoskeleton | Cytoplasm | Cytosol |
| Formin 2 | FMN2 | 26186194 | Cytosol | Nucleus | PM | Cytosol | Cytosol |
| GRB2 associated binding protein 1 | GAB1 | 26186194 | Cytosol | ER | PM |  | Cytosol |
| Serpin family B member 9 | SERPINB9 | 10477277 | Cytosol | Extracellular | Nucleus | Cytoplasm | Cytosol |
| TNF receptor associated factor 2 | TRAF2 | 22195745 | Cytosol | ER | PM | Cytoplasm | Cytosol |
| Androgen receptor | AR | 9535906 | Nucleus | Cytosol | Cytoskeleton | Nucleus | Nucleus |
| Atrophin 1 | ATN1 | 9535906 | Nucleus |  |  | Nucleus | Nucleus |
| Cyclin dependent kinase 11A | CDK11A | 9115219 | Nucleus | Cytosol | Cytoskeleton | Nucleus | Nucleus |
| Cyclin dependent kinase 11B | CDK11B | 9632733 | Nucleus |  |  | Nucleus | Nucleus |
| AT-rich interaction domain 4B | ARID4B | 26186194 | Nucleus |  |  | Nucleus | Nucleus |
| Baculoviral IAP repeat containing 3 | BIRC3 | 22195745 | Nucleus | Cytosol | Mito | Nucleus | Nucleus |
| CCAAT/enhancer binding protein beta | CEBPB | 11684016 | Nucleus | Extracellular | Cytosol | Nucleus | Nucleus |
| Vac14, PIKFYVE complex component | VAC14 | 25416956 | Endosome | Golgi | ER | Endosome | Endosome |

Abbreviation: Ѱ：From CASP 1 (Gene ID: 834) interaction in GENE of NCBI (National Center of Biotechnology Information); PM: plasma membrane; ER: endoplasmic reticulum; Mito: mitochondrion; #: Compartments subcellular location database; &: UniProtKB/Swiss-Prot location database; *: Summary data to see figure 2, and the red is confirmed by both database 1 and database 2.

Table S4. Danger signals involved in inflammasome activation.

A: Cytoplasmic danger signals.

| **Cytoplasmic danger signals** | | **PMID** |
| --- | --- | --- |
| **Extracellular signals** | |  |
|  | ATP | 19034348 |
|  | Degraded extracellular matrix (biglycans, hyaluronan) | 19605353/ 19258328 |
|  | Uric acid crystals | 25813103 |
|  | Beta amyloids | 18604209 |
| **Intracellular signals** | |  |
|  | Microbial double stranded DNA | 19158675/ 19158676 |
|  | Oxidized mitochondrial DNA released from stressed mitochondria | 22342844 |
|  | Reactive oxygen species (mitochondrial)/ NADPH | 21124315/ 18403674 |
|  | ER stress | 22278288 |
|  | Potassium efflux | 26814970 |
|  | Lysosomal damage | 18604214 |
|  | Pharmacological agents - Nelfinavir | 27462105 |
| **Proatherogenic stimuli** | |  |
|  | Proatherogenic lipids (LPC, LPA, oxyLDL) | 26037927/ 25705917 |

B: Nuclear danger signals

| **Nuclear viral infections** | | **PMID** |
| --- | --- | --- |
| **Herpes virus genomes** | |  |
|  | Kaposi's sarcoma-associated herpes virus (KSHV) | 21575908 |
|  | Epstein-Barr virus (EBV) | 23720728 |
|  | Herpes simplex virus type-1 (HSV-1) | 23427152 |
| **Pharmacological agents** | |  |
|  | Doxorubicin | 25501827 |
| **Proinflammatory stimuli** | |  |
|  | TNF-1α | 9726961 |

Figure S1. In coronary artery disease, the signal pathway of caspase-1 cytosolic, nuclear and extracellular substrates.

A. Upregulated caspase-1 cytosolic substrates in coronary heart disease induce ER stress and apoptosis.


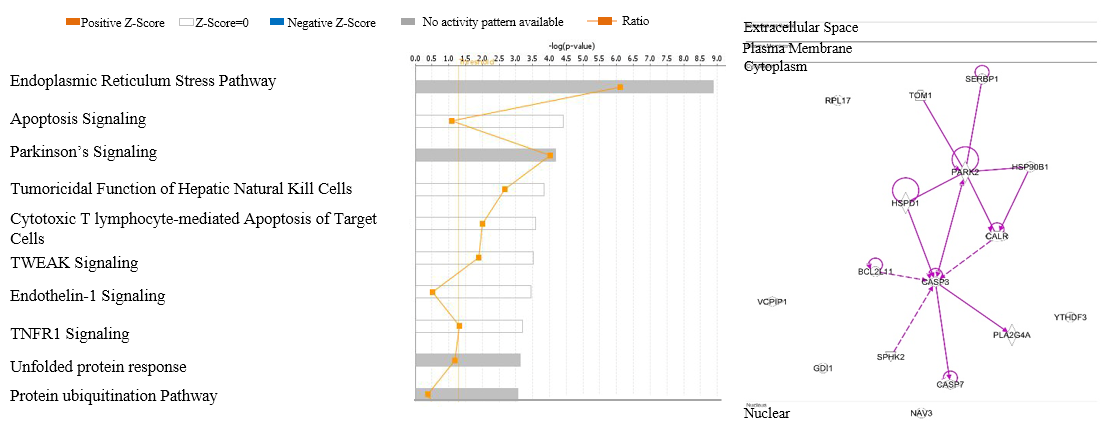


B. Upregulated caspase-1 nuclear substrates in coronary heart disease induce cell death, chromatin replication and inflammation.


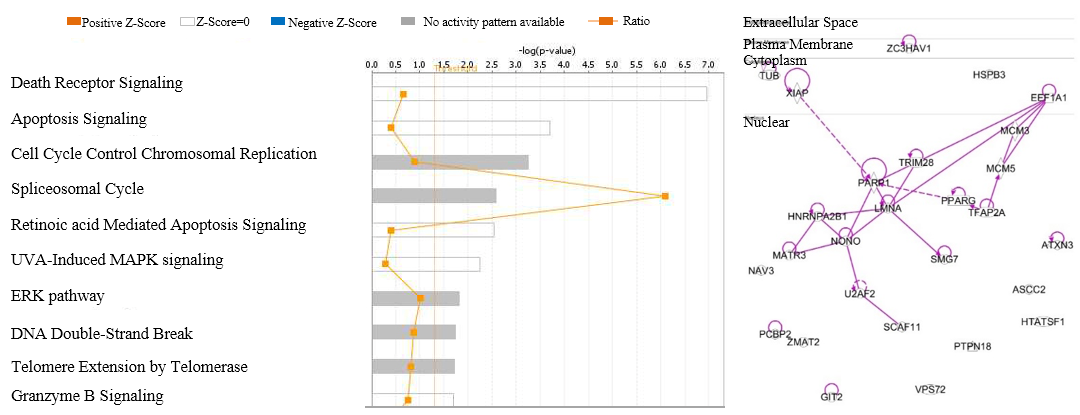


C. Upregulated caspase-1 extracellular substrate-related pathways mediate inflammation.


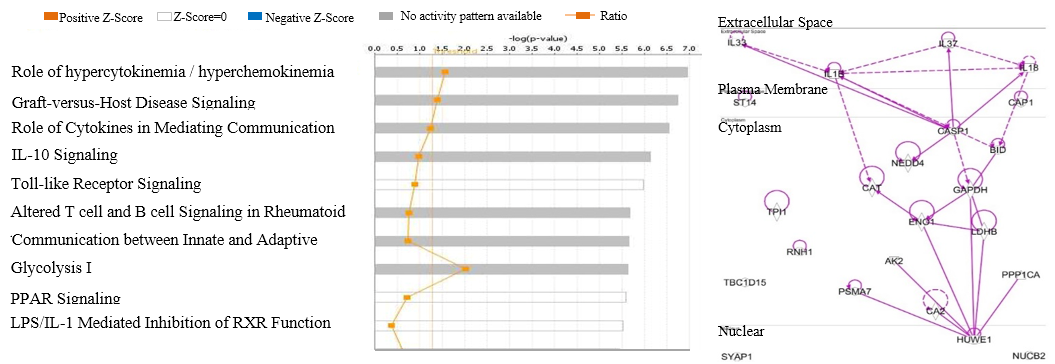

Supplement: Additional file 1: Table S1. — Analysis of 21 experimentally verified subcellular markers to confirm the reliability of two different databases. Table S2. 114 experimentally identified caspase-1 substrates are localized in various organelles including nucleus and secreted extracellularly. Table S3. 38 experimentally verified caspase-1 interaction proteins are localized in various intracellular organelles. Table S4. Danger signals involved in inflammasome activation. Figure S1. In coronary artery disease, the signal pathway of caspase-1 upregulated cytosolic, nuclear and extracellular substrates. (DOCX 828 kb) [file 13045_2016_351_MOESM1_ESM.docx]
